# [Expression of Concern] Reduced migration of Ishikawa cells associated with downregulation of aquaporin-5

**DOI:** 10.3892/ol.2026.15889

**Published:** 2026-09-30

**Authors:** Xiu Xiu Jiang, Kai Hong Xu, Jun Yan Ma, Yong Hong Tian, Xiao Yan Guo, Jun Lin, Rui Jin Wu

Oncol Lett 4: 257–261, 2012; DOI: 10.3892/ol.2012.738

Following the publication of this paper, it was drawn to the Editor's attention by a concerned reader that, regarding the images of AQP5-expressing Ishikawa cells shown in Fig. 1 on p. 259, the ‘Blank/72 h’ and ‘Control/72 h’ data panels contained an overlapping section of data, suggesting that these images had been derived from the same original source even though the results from differently performed experiments were intended to have been portrayed.

The authors have been contacted by the Editorial Office to offer an explanation for the apparently erroneous assembly of the data in Fig. 1, and we are awaiting their response. Owing to the fact that the Editorial Office has been made aware of potential issues surrounding the scientific integrity of this paper, we are issuing an Expression of Concern to notify readers of this potential problem while the Editorial Office continues to investigate this matter further.

